# Pharmacopuncture and joint movement manual therapy for post-traumatic phalangeal osteoarthritis: A case report

**DOI:** 10.1097/MD.0000000000027081

**Published:** 2021-09-24

**Authors:** Jinwoong Lim, Kyu-hyeong Kim, Sang-Hoon Shin, Seung-Hwan Lee, Jiyeon Lee, Hae In Ahn, NamKwen Kim

**Affiliations:** a Department of Acupuncture and Moxibustion, Mokhuri Neck and Back Hospital, Seoul, Republic of Korea; b Department of Clinical Korean Medicine, College of Korean Medicine, Graduate School, Kyung Hee University, Seoul, Republic of Korea; c You One Clinic, Seoul, Republic of Korea; d Tong-In Korean Medicine Clinic, Seoul, Republic of Korea; e Department of Sasang Constitutional Medicine, Kyung Hee University Hospital at Gangdong, Seoul, Republic of Korea; f Guideline Center for Korean Medicine, National Institute for Korean Medicine Development, Seoul, Republic of Korea; g Pusan National University Graduate School of Korean Medicine, Pusan, Republic of Korea.

**Keywords:** case report, joint movement manual therapy, phalangeal arthritis, pharmacopuncture, post-traumatic osteoarthritis

## Abstract

**Introduction:**

Post-traumatic osteoarthritis (PTOA) is a type of osteoarthritis that develops after ligament injury, meniscus injury, or fracture. Currently, there is no specific treatment approved for PTOA. This report describes the case of a 38-year-old man who suffered from PTOA of the right second distal interphalangeal (DIP) joint after practicing judo.

**Patient concerns:**

He visited the author's clinic at 3 months after the onset of symptoms. Symptoms included pain, limited motion, and joint enlargement of the right second DIP joint.

**Diagnosis:**

Partial tear of the ulnar collateral ligament of the DIP was revealed by magnetic resonance imaging. As the symptoms appeared after the traumatic event, PTOA was diagnosed.

**Interventions:**

Intra-articular hominis placenta pharmacopuncture and joint movement manual therapy were performed on each visit. Altogether, 10 sessions were performed until the symptoms improved remarkably.

**Outcomes:**

Visual analogue scale score (VAS) for pain; Quick Disabilities of the Arm, Shoulder, and Hand score (QuickDASH); joint circumference; and range of motion showed improvements at the end of the treatment. VAS decreased from 8.4 to 0.4, QuickDASH decreased from 44 to 13, joint circumference decreased from 5.5 to 5.4 cm, and range of motion was almost recovered, which was measured by the photographs.

**Lessons:**

There are not enough studies on phalangeal joint PTOA and its treatment. This case suggests pharmacopuncture and joint movement manual therapy as treatment options for phalangeal PTOA.

## 1 Introduction

Post-traumatic osteoarthritis (PTOA) is defined as osteoarthritis after injury such as fracture, acute ligament sprain, and cartilage damage.^[1]^ PTOA is known to recover after 2–3 months.^[2]^ However, symptoms persisting beyond 3 months need clinical attention. In clinical practice, symptoms persisting for more than 6 months indicate chronic PTOA.^[3]^ Treatments for the prevention of chronic PTOA and for managing the symptoms of PTOA include anti-inflammatory drugs, light exercise, and lifestyle consultation. However, there is no specific approved treatment.^[3]^

In Korean medicine, various treatment modalities such as acupuncture, moxibustion, and cupping have been shown to be effective for musculoskeletal diseases. A case study reported the effect of bee venom pharmacopuncture on acute PTOA of the elbow joints.^[4]^ However, there is insufficient evidence to support the use of these treatments for PTOA.

In this case report, we describe the case of a patient with PTOA who successfully improved after several sessions of pharmacopuncture and manual therapy.

## 2 Case report

**Figure 1:**
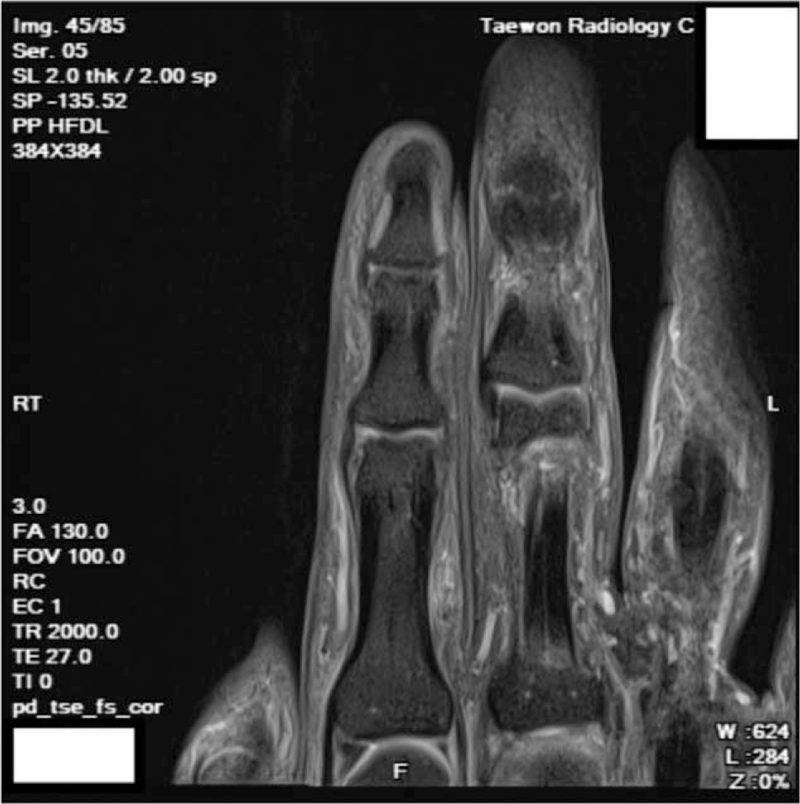
Proton density magnetic resonance image of the right second distal interphalangeal joint.

A 38-year-old man experienced pain with limited range of motion in the right second finger while practicing judo in October 2019. He visited a Korean medicine clinic; however, after 10 sessions of Korean medicine treatment including acupotomy, the symptoms deteriorated. Magnetic resonance imaging of the right hand at an orthopedic clinic on January 8, 2020, revealed partial tear of the ulnar collateral ligament of the distal interphalangeal (DIP) joint of the second finger (Fig. 1). He visited Kim's clinic with joint enlargement, pain, and persistent limited range of motion on January 22, 2020.

### 2.1 Treatments

Hominis placenta (C1-JH, Jaseng Wonoe Tangjunwon, Namyangju, Korea) was injected into the joint and manual therapy for joint mobilisation was performed on each visit. Altogether, 10 treatment sessions were performed until remarkable improvements were observed in the symptoms. The patient visited the clinic once every 1–2 weeks for 3 months.

**Figure 2:**
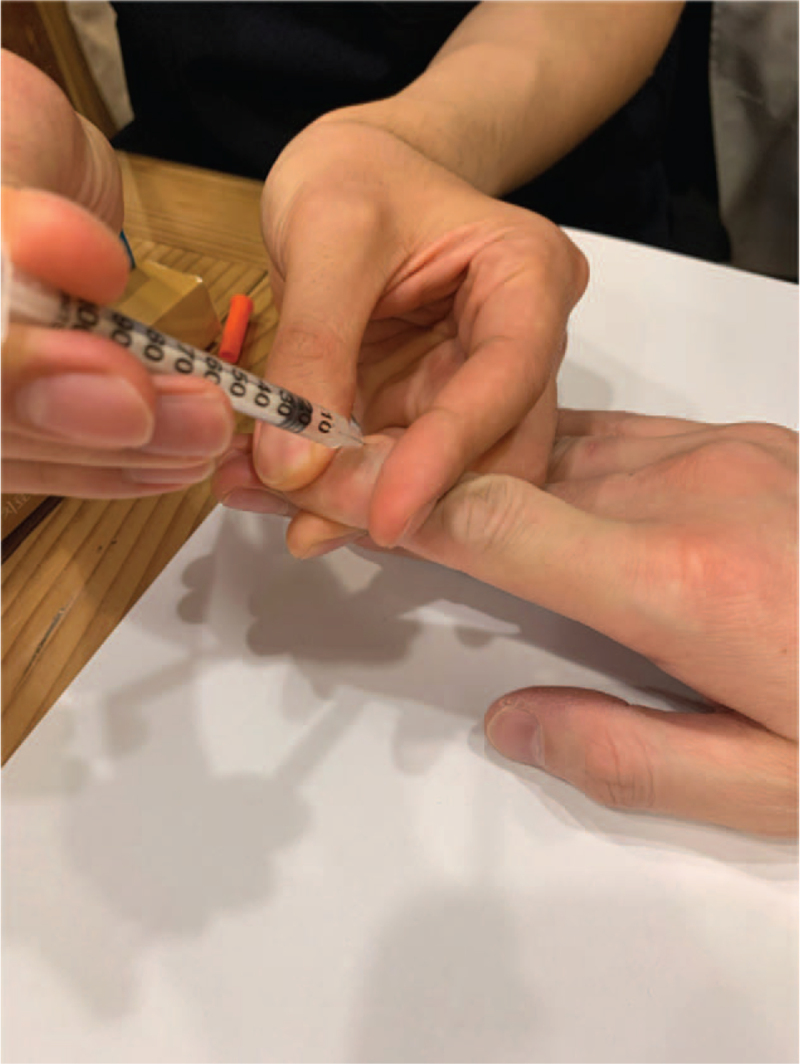
Intra-articular pharmacopuncture injection to the second distal interphalangeal joint.

**Figure 3:**
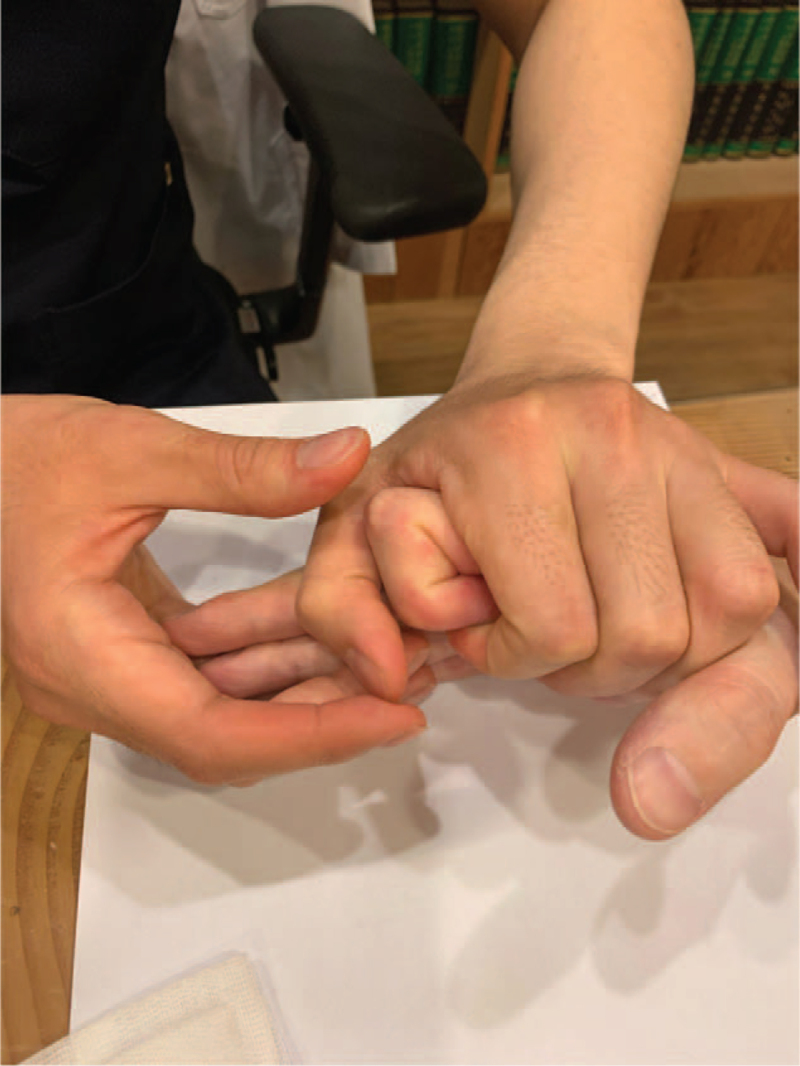
Hyperflexion of the second distal interphalangeal joint.

**Figure 4:**
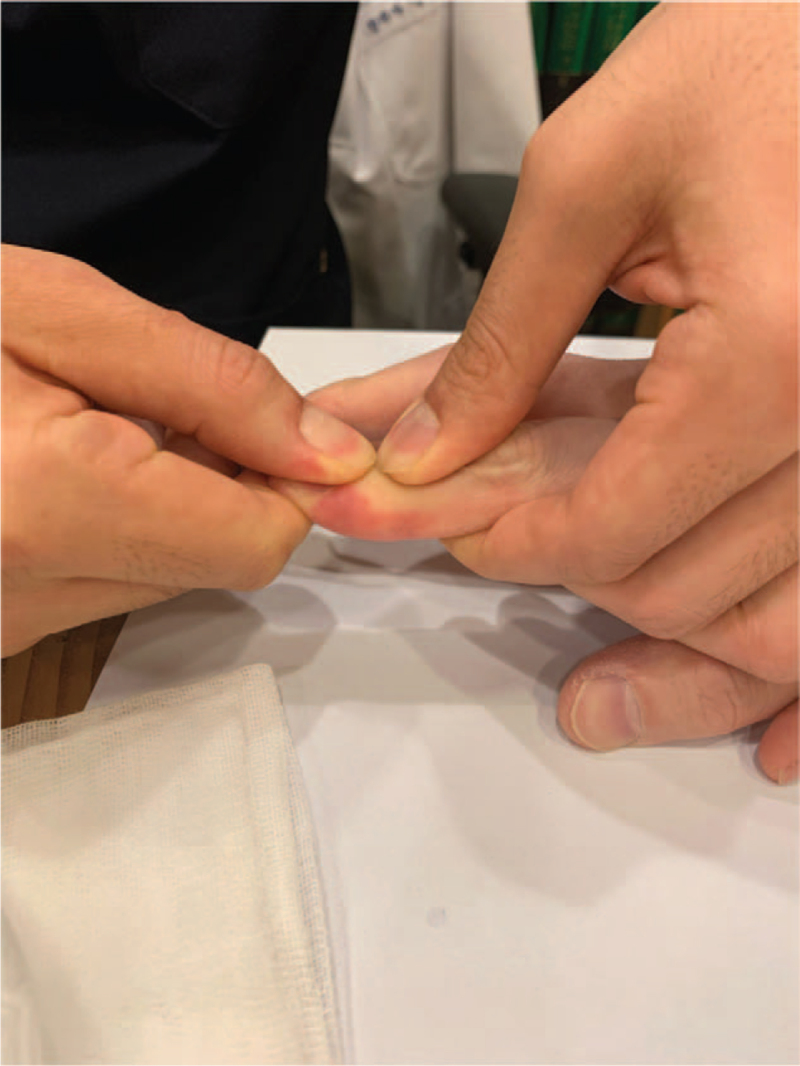
Hyperextension of the second distal interphalangeal joint.

Initially, the practitioner flexed the joint slightly and injected up to 0.4 mL of Hominis placenta into the intra-articular region through both the collateral ligaments (Fig. 2). Subsequently, the practitioner performed hyperflexion of the joint over the active range of motion and maintained it for 8–10 seconds (Fig. 3), followed by hyperextension of the joint for 8–10 seconds (Fig. 4). The practitioner repeated hyperextension and hyperflexion of the joint six times at each session.

### 2.2 Results

#### 2.2.1 Pain visual analogue scale (VAS)

Pain visual analogue scale (VAS) was primarily used to investigate the severity of pain. The patient reported his pain VAS score of the DIP joint before every treatment session. The pain VAS score was 8.4 at the first visit, 6.9 at the second visit, 4.8 at the fifth visit, and 0.4 at the final visit, thereby indicating decrease (Table 1).

**Table 1:** Measurements results of the patient.

Visit	1st	2nd	3rd	4th	5th	6th	7th	8th	9th	10th
date	’20/1/22	1/28	1/31	2/7	2/17	2/25	3/3	3/17	3/31	4/17
VAS	8.4	6.9	6.6	5.9	4.8	3.9	2.8	1.2	0.6	0.4
QuickDASH	44	–	–	–	–	31	–	–	13	–
Joint circumference, cm	5.5	5.5	5.5	5.5	5.5	5.5	5.4	5.4	5.35	5.4

QuickDASH = quick disabilities of the arm, shoulder, and hand questionnaire, VAS = visual analogue scale.

#### 2.2.2 Range of motion

**Figure 5:**
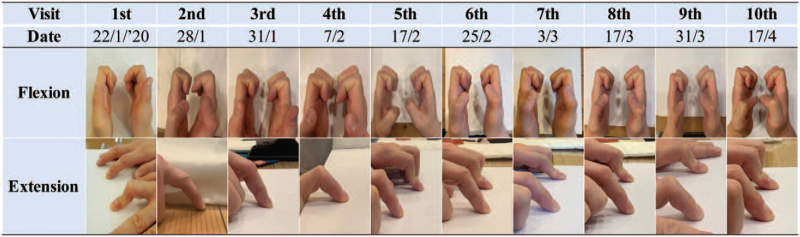
Change of the range of motion of the second distal interphalangeal joint.

Range of motion was investigated by obtaining photographs of the finger at every visit (Fig. 5). An active range of joint motion was observed. Unfortunately, the exact degree of motion was not measured. However, it is evident from Fig. 5 that the flexion and extension movements showed improvements after the fourth visit and gradually improved thereafter. The flexion was almost equivalent to that of the left second DIP joint and the extension could be considered satisfactory, although it was slightly different from that of the left second DIP joint at the final visit.

#### 2.2.3 Joint circumference

We measured the thickest part of the DIP joint at every visit. At the first visit, it was 5.5 cm, which persisted until the seventh visit. However, the circumference decreased after the seventh visit to 5.4 cm when the VAS score was 1.2 (Table 1).

#### 2.2.4 Quick disabilities of the arm, shoulder, and hand (QuickDASH) questionnaire

The QuickDASH questionnaire is used to specifically measure the upper-extremity symptoms and disability.^[5]^ It consists of 11 items derived from the 30-item DASH questionnaire. The Korean version of QuickDASH was verified in a previous study.^[6]^ The QuickDASH score ranges from 0 to 100, with a score of 100 representing absolute disability. The QuickDASH score was measured once a month in this study. It was 44 at the first visit, 31 at the sixth visit, and decreased to 13 at the ninth visit (Table 1). Thus, the joint disability was initially moderate and improved to a status of low disability after 2 months of treatment.

#### 2.2.5 One-year follow-up

**Figure 6:**
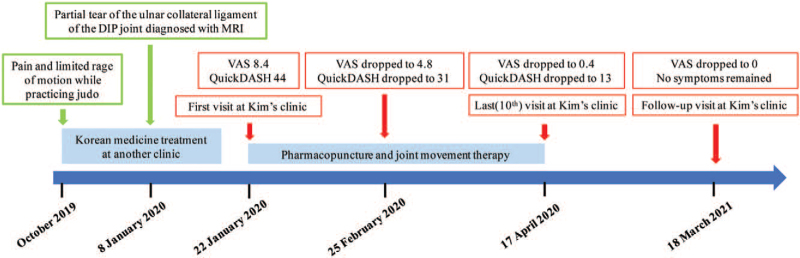
Timeline of the case. DIP = distal interphalangeal, MRI = magnetic resonance image, QuickDASH = quick disabilities of the arm, shoulder, and hand questionnaire, VAS = visual analogue scale.

The patient revisited the clinic on March 18, 2021. The pain VAS was 0, and no symptoms remained (Fig. 6).

### 2.3 Ethics approval

This study was a retrospective chart review and approved for exemption from deliberation by the Institutional Review Board of Mokhuri Neck and Back Hospital (MHNBH-IRB-20002).

## 3 Discussion

PTOA is a type of osteoarthritis that accounts for approximately 12% of the cases of symptomatic osteoarthritis in the USA.^[7]^ Unlike other forms of osteoarthritis, it is more common in young patients,^[7]^ and knee and ankle joints have been studied more extensively than other joints.^[1]^ Vigorous inflammatory response after injury is reported as a mechanism of development of PTOA^[2]^ and its chronic stage.^[3]^ Preventing the onset and progression of PTOA is important because PTOA affects the long-term health and quality of life.^[8]^ Nonsurgical treatments for PTOA include nonsteroidal anti-inflammatory drugs, intra-articular glucocorticoids, and physical therapy, which can be generally applied to osteoarthritis.^[3,9,10]^ However, management of the chronic stage of PTOA has not been clearly established.

We have described a case of phalangeal PTOA that occurred after practicing judo. Although epidemiology and characteristics of phalangeal PTOA have not been reported, we improved the symptoms of phalangeal PTOA with pharmacopuncture and joint manual therapy.

Traditionally, Hominis placenta pharmacopuncture has been applied to treat various diseases.^[11]^ Several studies have reported the possible benefits of pharmacopuncture for musculoskeletal diseases.^[12,13]^ Hominis placenta contains bioactive substances such as polydeoxyribonucleotides, amino acids, and peptides.^[14]^ The mechanism by which pharmacopuncture affects osteoarthritis is not clear. However, an experimental study reported that cells positive for tumor necrosis factor-α, interleukin-1β, and interleukin-6, which are critical in mediating the destruction of the cartilage and bone in arthritis; were decreased after Hominis placenta injection in rats with adjuvant-induced polyarthritis.^[15]^ In the present case report, intra-articular injection of Hominis placenta showed clinical improvements in PTOA, and it can be assumed that the anti-inflammatory effect of Hominis placenta pharmacopuncture helped alleviate the symptoms of PTOA.

In manual therapy, a practitioner uses his/her hands to reduce pain, mobilize a joint, and improve the physical condition of patients.^[16]^ Manual therapy has been recommended for various musculoskeletal diseases such as neck pain,^[17]^ shoulder impingement,^[18]^ and knee arthritis.^[19]^ However, manual therapy methods can differ according to countries and diseases and manual therapy for phalangeal disorders has rarely been reported. We performed hyperextension and hyperflexion of the second DIP joint of the patient to mobilize the joint and to reduce pain. Reduced joint stiffness and improved physical function of the joint were observed after the therapy. Therefore, this technique can be considered effective in preventing the aggravation of phalangeal PTOA and is recommended for other phalangeal joint disabilities.

Currently, there are not enough studies or reports on phalangeal joint PTOA and its treatment. In the present case, the patient suffered from PTOA of the second DIP joint. Clinically, it was about to progress to the chronic stage. Improvements were observed through several measurements after 10 treatment sessions, and no severe adverse events were reported. Small joints such as the interphalangeal joint have limited treatment options compared with other joints. This case report showed the possible advantages of pharmacopuncture and manual therapy as practical treatments for disorders of small joints. Therefore, these treatments can be considered feasible options to manage PTOA of the phalangeal joints. Future studies need to focus on the effectiveness and optimal amount of treatment along with the possible mechanisms of action.

## Author contributions

**Conceptualisation:** Kyu-hyeong Kim

**Funding acquisition:** Hae In Ahn, NamKwen Kim

**Investigation:** Kyu-hyeong Kim, Sang-Hoon Shin, Seung-Hwan Lee, Jiyeon Lee

**Methodology:** Jinwoong Lim

**Writing – original draft:** Jinwoong Lim

**Writing – review & editing:** Hae In Ahn, NamKwen Kim
